# Beyond Borders—Tackling Neglected Tropical Viral Diseases

**DOI:** 10.3390/tropicalmed11010029

**Published:** 2026-01-21

**Authors:** Jelena Prpić

**Affiliations:** Croatian Veterinary Institute, 10000 Zagreb, Croatia; balatinec@veinst.hr

Neglected tropical diseases (NTDs) comprise a diverse group of infections that disproportionately affect impoverished populations in tropical and subtropical regions. Among them, viral NTDs, including dengue, Zika, chikungunya, yellow fever, West Nile virus, Oropouche virus, and other emerging arboviruses, represent a growing global health concern due to their expanding geographic distribution, increasing incidence, and complex interactions with environmental and socioeconomic factors [1,2,3]. More than one hundred arboviruses are known to infect humans, and many continue to emerge or re-emerge as ecological and climatic conditions shift. According to the World Health Organization (WHO), nearly 3.9 billion people now live in areas at risk for *Aedes*-borne arboviral infections, and the frequency and magnitude of outbreaks have increased markedly over the past decade, driven by climate variability, rapid urbanization, and intensified human mobility [2,3,4].

The WHO’s Global Arbovirus Initiative underscores the urgency of coordinated global action, emphasizing that arboviruses such as dengue, Zika, chikungunya, and yellow fever are expanding into new ecological niches and placing unprecedented strain on public health systems. Climate change is accelerating vector expansion into temperate regions, including parts of Europe, where *Aedes albopictus* and *Aedes aegypti* have become increasingly established [5]. These shifts are reshaping arboviral transmission dynamics and challenging traditional assumptions about the geographic limits of tropical viral diseases.

Beyond environmental drivers, structural vulnerabilities, including inadequate vector control, limited access to diagnostics, fragile health systems, and insufficient community engagement, continue to exacerbate the burden of viral NTDs [3,4]. Surveillance gaps allow silent transmission to persist for years before major outbreaks are detected, particularly in low-resource settings. Although advances in biomedical research have improved diagnostic and surveillance capacities, many endemic regions still lack the laboratory infrastructure needed for timely detection and response. Interdisciplinary research increasingly highlights the importance of integrating community-based strategies with scientific innovation to strengthen outbreak preparedness and improve public health outcomes.

Addressing these challenges requires coordinated, cross-border, and interdisciplinary approaches that integrate epidemiology, vector biology, diagnostics, clinical research, and community engagement. As global mobility intensifies and climate change accelerates, the need for adaptive and collaborative public health responses has never been greater.

In this Special Issue of Tropical Medicine and Infectious Diseases, “Beyond Borders—Tackling Neglected Tropical Viral Diseases”, we invited contributions that explore innovative strategies, research advances, and collaborative efforts aimed at understanding and controlling neglected tropical viral diseases. Eight articles, spanning epidemiological analyses, diagnostic innovation, seroprevalence studies, systematic reviews, and immunological insights, were published.

Costa-Ribeiro et al. assessed the impact of mass dengue vaccination on disease incidence in Paraná, Brazil, using an interrupted time series approach [6]. Their findings highlight the potential of large-scale immunization programs to reduce dengue burden, while also emphasizing the importance of continuous monitoring and adaptive public health strategies.

Reigoto et al. explored the use of limited near-infrared spectroscopy data combined with machine learning to detect Zika virus infection in *Aedes aegypti* mosquitoes [7]. This innovative vector-based diagnostic approach demonstrates the feasibility of rapid, non-destructive surveillance tools, particularly valuable in resource-limited settings.

Chandrasena et al. conducted a systematic review and meta-analysis of dengue-related knowledge, attitudes, and practices (KAPs) among the general public in Sri Lanka from 2000 to 2023 [8]. Their synthesis reveals persistent gaps in community awareness and preventive behaviors, underscoring the need for sustained, culturally tailored health education initiatives.

To et al. evaluated baseline IgG seroprevalence of multiple arboviruses in Liberia using a multiplex immunoassay [9]. Their findings demonstrate widespread exposure to several arboviruses, highlighting the silent circulation of these pathogens and the need for integrated serological surveillance in West Africa.

Amaral et al. performed a systematic review and meta-analysis examining the association between chikungunya fever and rheumatoid arthritis [10]. Their results suggest that chikungunya infection may trigger or exacerbate chronic inflammatory joint disease, emphasizing the long-term clinical consequences of arboviral infections.

Melebari et al. characterized dengue virus serotypes among patients presenting with dengue fever in Makkah, Saudi Arabia [11]. Their identification of multiple co-circulating serotypes provides essential information for anticipating outbreaks and guiding clinical and public health preparedness in a region marked by intense international travel.

He et al. reviewed the mechanisms by which bunyaviruses suppress interferon responses and summarized current antiviral strategies [12]. Their work highlights the sophisticated immune evasion tactics of bunyaviruses and identifies potential therapeutic targets.

Finally, Ríos-Bracamontes et al. analyzed national surveillance data from Mexico (2020–2024) to identify factors associated with in-hospital dengue mortality [13]. Their findings provide critical insights into clinical predictors of severe disease and underscore the importance of early recognition and optimized case management.

The articles presented in this Special Issue collectively underscore that neglected tropical viral diseases remain among the most dynamic and challenging threats to global health. Despite decades of scientific progress, the persistent and often widening gaps in surveillance, diagnostics, vector control, and clinical management reveal how deeply these diseases are intertwined with structural inequities. The research showcased here demonstrates that scientific innovation alone is not sufficient; rather, it must be paired with sustained investment in public health infrastructure, community engagement, and international cooperation.

A recurring theme across the contributions is the importance of early detection and rapid response. Whether through innovative vector-based diagnostic tools, multiplex serological assays, or improved epidemiological modeling, the ability to identify outbreaks before they escalate is essential. Strengthening laboratory capacity and integrating novel technologies into routine surveillance can help countries detect silent transmission, monitor serotype shifts, and anticipate severe disease outcomes.

Another critical insight emerging from this Special Issue is the long-term clinical and societal impact of arboviral infections. Studies examining chronic sequelae, such as chikungunya-associated inflammatory disease, highlight the need for long-term patient follow-up and improved clinical guidelines. These findings challenge the misconception that arboviral infections are self-limiting and emphasize the importance of integrating post-acute care into national health strategies.

The contributions also reinforce the central role of community knowledge, behavior, and trust in shaping outbreak trajectories. Persistent gaps in public awareness, as documented in Sri Lanka and other endemic regions, demonstrate that effective vector control and prevention strategies depend on culturally informed, community-centered approaches. Public health messaging must be continuous, context-specific, and responsive to local perceptions and barriers.

Importantly, the Special Issue highlights how global forces, climate change, urbanization, migration, and international travel are reshaping arboviral ecology. These forces transcend national borders, making unilateral approaches insufficient. Cross-border data sharing, harmonized surveillance systems, and coordinated vector control strategies are essential to prevent the spread of arboviruses into new regions and to mitigate the impact of outbreaks in highly interconnected societies.

Finally, the collective body of work presented here illustrates the value of interdisciplinary collaboration. Virologists, clinicians, entomologists, epidemiologists, data scientists, and public health practitioners each contribute essential perspectives. When these disciplines converge, they generate insights that are greater than the sum of their parts. This Special Issue demonstrates that tackling neglected tropical viral diseases requires not only scientific excellence but also a shared commitment to equity, solidarity, and global health security.

As we look ahead, it is clear that the challenges posed by viral NTDs will continue to evolve. However, the research featured in this Special Issue offers a roadmap for progress, one grounded in innovation, collaboration, and a recognition that no community should be left behind. Continued investment in research, surveillance, and international partnerships will be essential to reduce the burden of these diseases and to build a more resilient and equitable global health landscape.

## Data Availability

Not applicable.
